# Delivering albumin-bound paclitaxel across the blood-brain barrier for gliomas

**DOI:** 10.18632/oncotarget.28018

**Published:** 2021-12-07

**Authors:** Andrew Gould, Daniel Zhang, Víctor A. Arrieta, Roger Stupp, Adam M. Sonabend

**Keywords:** glioblastoma, focused ultrasound, blood brain barrier, paclitaxel

In Zhang et al. we report a novel means of delivering paclitaxel across the blood-brain barrier (BBB) using low-intensity pulsed ultrasound (LIPU) and microbubbles. This study is the basis for repurposing albumin-bound paclitaxel (PTX) for the treatment of glioblastoma (GBM)[1], an established chemotherapy agent that has demonstrated remarkable antitumor activity in preclinical GBM models. GBM is the most common and malignant primary brain cancer in adults, an incurable disease with dismal prognosis [2]. A contributor to this poor outcome for GBM patients is that most cytotoxic drugs (including PTX) are unable to penetrate across the BBB and reach therapeutically relevant concentrations in the brain parenchyma. This limitation of conventional systemic delivery of many chemotherapy drugs allows residual tumor cells to escape the treatment by “hiding” behind the intact BBB [3]. LIPU with the intravenous injection of microbubbles is a promising new technology that seeks to overcome this limitation by temporarily and reversibly opening the BBB. This technique works by exposing inert bubbles of gas to an alternating acoustic pressure. The rapid expansion and contraction of these bubbles generates a mechanical force that renders vessel walls transiently permeable and improves drug delivery [4].

Preclinical and human studies have shown that this technique is safe, well tolerated, and able to increase brain tissue concentrations of drugs like temozolomide and carboplatin [5–7]. Newer generation ultrasound probes have also been developed that can be implanted directly into a patient’s skull and the sound waves can bypass the bone as a window is created in the skull for the implant [7, 8]. These devices are an interesting alternative to transcranial technologies, which require multiple ultrasound waves to penetrate through the skull, a process that is often guided by MRI. Furthermore, implanted probes allow for easy and unchanging placement of the ultrasound field over repeat treatments, which could take place in outpatient settings. This promising new technology sets the stage for Zhang et al. to re-contextualize the use of PTX for glioma therapy. Traditionally employed against many solid tumors, PTX has not been successful in the treatment of GBM as previous formulations have demonstrated limited brain penetrance and vehicle-associated peripheral nerve toxicity [9, 10].

Zhang et al. used Abraxane (ABX), a newer, albumin-bound formulation of PTX with lower rates of neuropathy compared to earlier iterations [11]. Early experiments by our group compared the biodistribution of PTX in mice treated with either ABX or Taxol (an older formulation of PTX that used neurotoxic Cremophor EL (CrEL) as a vehicle). As early as 45 minutes after treatment, mice treated with ABX demonstrated significantly higher brain to plasma PTX concentration compared to mice that were treated with Taxol. This phenomenon was maintained as late as 3 hours after treatment and was matched with consistently higher drug concentrations in the liver, indicating tissue penetration. This demonstrated that ABX had inherently favorable biodistribution compared to Taxol.

Whereas previous studies had mapped BBB disruption by the extravasation of contrast-enhancing agent gadolinium or Evan’s Blue, on Zhang et al. we visualized effective disruption by the accumulation of fluorescein. Experiments showed that disruption was highly localized to regions of the brain that were exposed to the ultrasound field, as judged by obvious fluorescence. In mice that underwent LIPU-BBB disruption and received treatment with PTX, there was a strong correlation between brain tissue concentrations of fluorescein and PTX, indicating that the disruption induced by the sonication field was also highly effective at facilitating drug delivery to the brain. While we showed that ABX by itself was markedly more permeable than Taxol in the absence of LIPU, it achieved approximately 4-fold higher concentrations in brain tissue that had undergone BBB disruption.

Toxic side-effects associated with chemotherapies are often a major limitation to treatments. In this study, ABX (at 12 mg/kg) in tandem with LIPU twice a week was well tolerated with minimal brain tissue damage. However, animals treated on an identical schedule with either Taxol or CrEL alone showed severe CNS toxicity, including necrosis and hemorrhage. This confirmed that most treatment-related damage was associated with the CrEL vehicle and not the drug itself. Ultimately, ABX delivered by ultrasound significantly extended the overall survival in an immunocompromised mouse model bearing MES83 human tumor cells compared to control groups that received ABX without ultrasound.

The use of fluorescein to map BBB disruption is also a choice worth noting. Earlier animal studies have employed toxic tracer dyes, like Evan’s blue, to judge disruption upon biopsy [12]. While simple and effective, the inherent toxicity of these substances limits the ability of researchers to judge the effectiveness of BBB disruption across a survival study. Obviously, such chemicals cannot be employed in human studies, where gadolinium-enhanced MRI has instead been used to image disruption hours after the treatment. By comparison, fluorescein is non-toxic and commonly used intraoperatively to map the neuro-vasculature and tumor margins. Fluorescein could therefore be used to judge effective BBB disruption in real time, to ensure maximal drug delivery to the tissue harboring infiltrative disease.

There are, however, limitations to this work that will need to be addressed in future studies. We noted that histological staining of the PDX tumors from their survival studies show extensive vascularization and minimal integration with the surrounding brain tissue. This contrasts to the infiltrative nature of GBM in humans, and likely resulted in some BBB disruption in the peritumoral tissue regardless of sonication treatment. Ongoing studies in patients include pharmacokinetic studies where we perform biopsies to determine drug quantification, particularly in peritumoral regions that maintain an intact BBB and are exposed to the sonication field (https://clinicaltrials.gov/ NCT04528680).

Through this work, we demonstrate the strong potential of LIPU technology, by recontextualizing the use of a potent drug that might otherwise go overlooked for the treatment of GBM. By overcoming the BBB as an obstacle to drug delivery, Zhang et al. open the door for a new gamut of therapies against brain cancers.
